# Correlation of Gas Permeability in a Metal-Organic Framework MIL-101(Cr)–Polysulfone Mixed-Matrix Membrane with Free Volume Measurements by Positron Annihilation Lifetime Spectroscopy (PALS) 

**DOI:** 10.3390/membranes3040331

**Published:** 2013-10-25

**Authors:** Harold B. Tanh Jeazet, Tönjes Koschine, Claudia Staudt, Klaus Raetzke, Christoph Janiak

**Affiliations:** 1 Institute of Inorganic and Structural Chemistry, Heinrich-Heine-University, 40204 Düsseldorf, Germany; E-Mail: harold.tanh.jeazet@uni-duesseldorf.de; 2 Institute for Materials Science, Faculty of engineering of the Christian-Albrechts-University, 24143 Kiel, Germany; E-Mail: tk@tf.uni-kiel.de; 3 Institute of Organic Chemistry and Macromolecular Chemistry, Heinrich-Heine-University, 40204 Düsseldorf, Germany

**Keywords:** gas separation, membranes, permeability, permselectivity, mixed-matrix membranes, metal-organic frameworks (MOFs), porosity, PALS

## Abstract

Hydrothermally stable particles of the metal-organic framework MIL-101(Cr) were incorporated into a polysulfone (PSF) matrix to produce mixed-matrix or composite membranes with excellent dispersion of MIL-101 particles and good adhesion within the polymer matrix. Pure gas (O_2_, N_2_, CO_2_ and CH_4_) permeation tests showed a significant increase of gas permeabilities of the mixed-matrix membranes without any loss in selectivity. Positron annihilation lifetime spectroscopy (PALS) indicated that the increased gas permeability is due to the free volume in the PSF polymer and the added large free volume inside the MIL-101 particles. The trend of the gas transport properties of the composite membranes could be reproduced by a Maxwell model.

## 1. Introduction

Membrane processes are an energy saving method for the separation of mixtures which occur in nearly all production processes in the chemical industry. Membrane based devices are much smaller and work at lower temperatures compared to conventional separation facilities with distillation, extraction or adsorption processes. Energy savings of up to 50% of the production costs can be reached by application of membrane technology [1]. The worldwide membrane market has a growth of approximately 10%–15% each year [2]. Membrane processes applied on industrial scale are natural gas treatment (removal of CO_2_ before the natural gas can be passed to the pipeline), hydrogen isolation and recovery (*i.e.*, in cracking processes) oxygen enrichment from air (medical devices) and nitrogen enrichment from air (used as protecting atmosphere for oxygen sensitive compounds) [3,4]. Other membrane based processes with fast growing market relevance are vapor recovery systems [5], monomer recovery units, e.g., ethylene/N_2_ or propylene/N_2_ separation [6,7], the dehydration of organic solvents and the removal of polar low molecular weight components in equilibrium reactions [8]. Commercially applied membrane materials are mostly polymers. Compared to inorganic materials they are cheap to produce and easy to process as flat sheet or hollow fiber membranes. Unfortunately, an important constraint in the development of polymer membranes for gas separation applications is the trade-off between permeability and selectivity, first demonstrated by Robeson and commonly called “Robeson upper bounds” [9,10,11]. 

The economics of membrane processes is mainly determined by the selectivity and the permeability as the most basic requirements for the choice of membrane materials [12]. With low selectivity the separation processes have to be multi-step which translates into higher operative complexity and costs. The permeability decides which membrane area or how many membrane modules are needed to realize the separation process. Membranes with a high permeability are needed for large-volume gas feed streams as in natural gas treatments or for pre-combustion O_2_/N_2_ separation.

**Figure 1:**
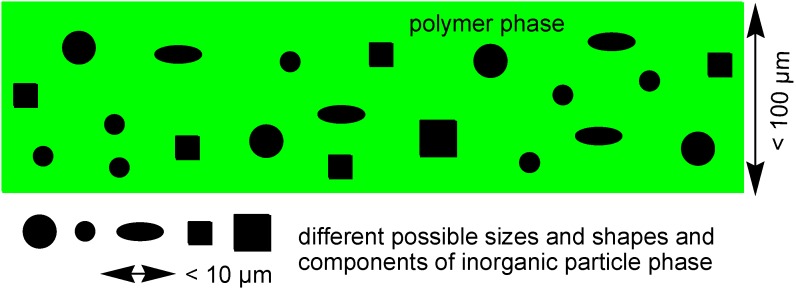
Schematic representation of a mixed-matrix membrane indicating different, also simultaneous possible sizes, shapes and components for the inorganic filler materials. 10 µm is a typical length scale of the particles, compared to the thickness of the polymer film of 100 µm.

Mixed-matrix membranes (MMMs) also called composite membranes consist of an inorganic or inorganic-organic hybrid material in the form of micro- or nanoparticles (discrete or dispersed phase) incorporated into a polymer matrix (continuous phase) (Figure 1) [12,13,14,15,16,17,18,19,20]. The use of two materials with different flux and selectivity allows to better design a gas separation membrane through the synergistic combination of easy processability of the polymer and superior gas separation performance of inorganic materials. Separation properties with MMMs can be above the Robeson upper bound. Porous inorganic fillers can counteract the trade-off between selectivity and permeability which is typical for pure polymer membranes. Different types of inorganic additives, impermeable and permeable ones, can be used as filler materials [12,15,16,17,18,21,22]. 

There is a strong interest in finding new membrane materials to meet the present and future requirements and challenges in membrane-based separation technologies [12]. The amount and distribution of free volume in polymeric membranes significantly determines the transport and separation properties. Recent improvement strategies are centered around the addition of porous particles, thus generating extra free volume. Metal-organic frameworks, MOFs are promising additives for MMMs since they offer various advantages over zeolites [23,24,25,26,27]. For example, ligands with a broad variety of functionalities are possible which enable MOFs to interact strongly with the polymer bulk material so that the formation of micro gaps between inorganic and organic phase, which cause loss in selectivity, can be avoided [15,16,17]. For the preparation of MMMs, a perfect interaction between the two components is extremely important in order to obtain materials with optimized separation properties. The literature on metal-organic frameworks in mixed-matrix membranes for gas separation has recently been reviewed [15,16].

Metal-organic frameworks (MOFs) or porous coordination polymers (PCPs) [28,29] have attracted tremendous attention over the past years [30,31,32,33,34,35,36]. This is due to their porosity, large inner surface area and tuneable pore sizes which leads to promising applications [30,37,38,39], such as gas adsorption and storage [40,41,42,43,44,45,46,47,48,49,50,51,52], gas and liquid separation [15,16,40,53,54,55,56,57,58], drug delivery [59,60,61], sensor technology [62,63,64], heterogeneous catalysis [65,66,67,68,69,70], hosts for metal colloids or nanoparticles [71,72] and recently water sorption for heat transformation [73,74,75,76,77]. 

Yet, understanding the separation properties of inorganic or MOF materials drops behind that of polymer membranes. A projectable inorganic material selection currently represents somewhat of a problem. Therefore, a standard and consistent approach is needed to identify the separation properties of inorganic or MOF materials. Intensive investigation is needed to assign the effects of particle size and geometry, particle pore size, and the polymer/particle interface needs to be examined. At the interface between the polymer and inorganic or MOF particles there is a further complicating phase boundary which makes prediction of the MMM performance difficult. Mechanical and permeability properties of polymers are strongly connected to free volume, which might be different at the boundary layer. This free volume can be either characterized from molecular dynamics simulations or from experiments, where a probe on atomic scale is needed. Positron annihilation lifetime spectroscopy (PALS) is a generally accepted method for investigation of free volume in polymers due to the so-called standard model developed by Tao and Eldrup [78,79]. This simple quantum mechanical model gives a direct relationship between pick-off lifetime of ortho-positronium and the size of the free volume holes. A clear correlation between changes of permeability and free volume was determined by PALS in polyphenylene oxide with different concentrations of hyperbranched polyesters (dendrimers) [80] and for 6FDA copolyimide films with nanoparticles [81]. PALS investigation of several Teflon AF 2400/silicon interfaces shows a clear interphase for spin-coated films [82]. 

Recently, polymer nanocomposite membranes with the MOF ZIF-8 as a filler and either polyimides (Matrimid, Ultem) [19,20,83] or PIM-1 (polymer of intrinsic microporosity) [84] as polymer matrix were tested for pure gas permeation. When Matrimid was used, PALS indicated that an increase in gas permeability is due to the free diffusion of the gas molecules through the ZIF-8 pores and a reduction in packing efficiency of the polymer [83]. In the case of ZIF-8/PIM-1 PALS indicated that the introduction of ZIF-8 nanoparticles into the PIM-1 matrix resulted in an increase in free volume which was assumed to arise from a combination of the filler cavities and of more loosely packed polymer chains at the boundary between ZIF-8 particles and the PIM-1 matrix [84].

Here, we report the gas separation properties of a MOF-MMM [85] made of MIL-101 [86] and polysulfone and analyze the free volume contributions with PALS.

## 2. Experimental

### 2.1. Materials

Chromium nitrate nonahydrate, Cr(NO_3_)_3_·9H_2_O (99%) and hydrofluoric acid (analysis grade) were obtained from Acros Organics. Benzene-1,4-dicarboxylic acid (H_2_BDC, 99%) was acquired from Aldrich. Dichloromethane (DCM, >99.9%), *N*,*N*ʹ-dimethylformamide (DMF, 99.9%) and ethanol (99.9%) were purchased from Prolabo. Polysulfone (PSF) Ultason S 6010 Natural was provided by BASF AG, Ludwigshafen, Germany. O_2_, N_2_, CO_2_ and CH_4_ gas were supplied by Air Liquide (Düsseldorf, Germany) and used as received (purity 99.99%). 

### 2.2. Synthesis of MIL-101

**Figure 2:**
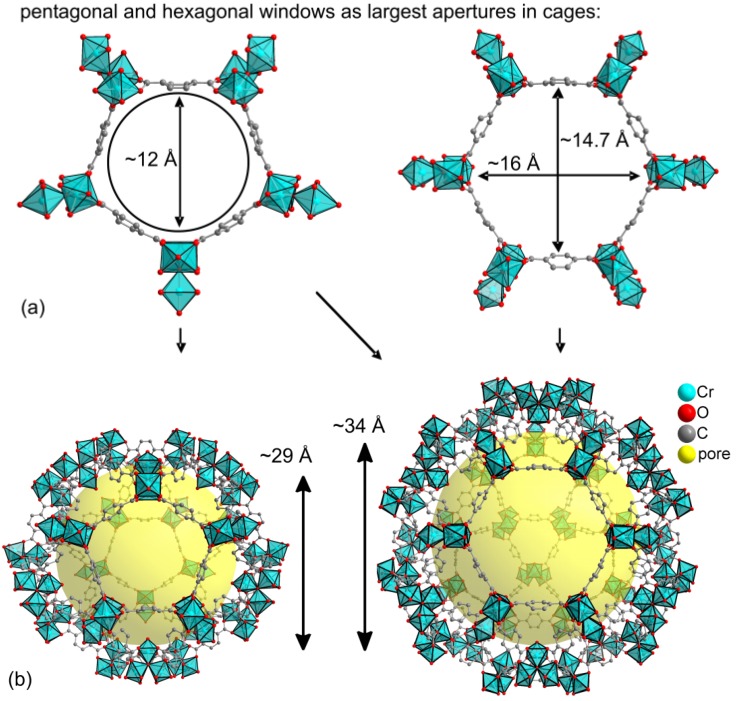
Building blocks for MIL-101, [Cr_3_(µ_3_-O)(F,OH)((BDC)_3_(H_2_O)_2_], generated from the deposited X-ray data file at the Cambridge Structure Database (CSD-Refcode OCUNAK) [86] using the program DIAMOND [87]. Trinuclear {Cr_3_O} building units and bridging benzene-1,4-dicarboxylate ligands form pentagonal and hexagonal rings (**a**) which are assembled into mesoporous cages (**b**). The yellow spheres in the mesoporous cages with diameters of 29 or 34 Å, respectively, take into account the van-der-Waals radii of the framework walls (water-guest molecules are not shown) [86]. The different objects in this figure are not drawn to scale.

{[Cr_3_(µ_3_-O)(X)((BDC)_3_(H_2_O)_2_]·25H_2_O} (BDC = benzene-1,4-dicarboxylate (terephthtalate), X = F or OH depending on synthesis conditions), MIL-101 (Figure 2) was synthesized according to the previously reported procedure [86]. A typical synthesis involves a solution containing chromium(III) nitrate Cr(NO_3_)_3_·9H_2_O (400 mg, 1 × 10^–3^ mol), 1 × 10^–3^ mol of hydrofluoric acid, benzene-1,4-dicarboxylic acid H_2_BDC (164 mg, 1 × 10^–3^ mol) in 5 mL H_2_O; the mixture is transferred to the Teflon line in a hydrothermal autoclave which is heated for 6 h at 210 °C and cooled afterwards slowly to room temperature over a time period of 8 h. The mixture was then isolated from the autoclave and the solid separated from the solution through centrifugation (4200 U/min for 50 min). A significant amount of terephthalic acid is present inside the pores and admixed with the MIL crystallites. The residual reactants and eventually the solvent needs to be removed especially from the pores in order to obtain a material with as high a porosity as possible. This washing and drying procedure is termed activation. To eliminate most of the carboxylic acid, the product was two times re-dispersed and stirred for 6 h in DMF (20 mL), two times in methanol (10 mL, 2 h) and one time in water (10 mL, 2 h) with separations from the respective washing solution by centrifugation. The final product was dried at room temperature.

The crystalline product (Figure S1 in the Supplementary Information) was positively identified as MIL-101 by powder X-ray diffraction (Figure S2). Activated MIL-101 samples have high pore volumes and surface areas close to samples in literature reports (Figure S3) [86]. The BET surface was determined to 2690 m^2^/g. Thermogravimetric analysis of as-synthesized MIL-101(Cr) (Figure S4) showed that up to 8% weight loss occurred until 120 °C, which is attributed to trapped water in the pores. Between 120 and 350 °C, 9% of material is lost. This may correspond to the loss of non-coordinated terephthalic acid from inside the pores. The MIL-101 framework starts to decompose above 350 °C. Activated MIL-101(Cr) (Figure S4) showed a 7% weight loss until 300 °C.

### 2.3. Preparation of Mixed-Matrix Membranes

**Figure 3:**
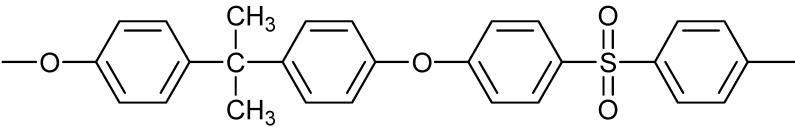
Polysulfone repeating unit.

The polymer (PSF) (Figure 3) was dissolved in dichloromethane (CH_2_Cl_2_) and the solution was filtered through a syringe filter (PTFE membrane, 0.45 µm pore size). 1.25, 1.88 and 2.5 wt % of polymer in CH_2_Cl_2_ were used. The MOF material (MIL-101) was added to the previously prepared polymer solution, and the obtained polymer-MIL dispersion was magnetically stirred for one week to achieve an intimate polymer-MIL mixture. MIL-101 was added to the polymer dispersion as 7.5, 14, 19 and 24 wt % relative to polymer. The SEM pictures in Figure 6 and also SEM pictures in reference [85] show, that the MIL crystallites remained intact through the long stirring period. Also X-ray powder diffraction (Figure S2 in the Supplementary Information) proved that stirring did not alter the crystallinity of MIL-101 particles. 

To achieve a homogeneous dispersion of the MOF particles the casting solution was treated for 30 min in ultrasonic bath (ELMA Transsonic 310, 35 Hz), afterwards it was stirred for 30 min again. This cycle was repeated three times. Before casting, the dispersion was kept under stirring for 30 more minutes. The dispersion was cast into metal rings, 7 cm in diameter, which were placed on a flat glass surface. All the casting equipment was placed on top of an adjustable table to assure horizontal alignment during the membrane formation. To prevent membrane contamination by dust particles during the evaporation of the solvent, funnels were used to cover the metal. A paper tissue covered the funnels to avoid contamination. This system also exerts some control on the evaporation rate. As soon as all solvent was evaporated, the membrane was removed from the metal ring and the glass surface by flushing the ring with distilled water. The membrane was finally dried in a vacuum oven at 120 °C and 80 mbar overnight. The pure polymer membranes were dried in the same way. 

### 2.4. Membrane Characterization Methods

Scanning electron microscopy (SEM) images were acquired by coating the membranes cross-sections with gold. The cross sections were obtained by breaking the membranes which were frozen in liquid nitrogen. The coated membrane samples were then imaged using an ESEM Quanta 400 FEG SEM equipped with a secondary electron (SE) detector and operated at 20 keV.

The polymer matrix does not alter the crystalline pattern of MIL-101 (Figure S2). Thermogravimetric analyses of activated MIL-101(Cr)/polysulfone membranes (Figure S4) showed almost no weight loss up to 350 °C, indicating that the casting solvent was not trapped in the pores of the MIL-101 framework. The PSF polymer decomposed above 500 °C (Figure S4).

### 2.5. Gas Permeation Experiments

Single-gas permeabilities were evaluated for O_2_, N_2_, CO_2_ and CH_4_ using the permeation cell described elsewhere [88]. Before affixing the membrane to the permeation cell, the thickness of the membrane was measured on 10 different points using a micrometer screw. The gas permeation measurements were performed at steady state conditions using the pressure rise method at 30 °C. The membrane is placed into the cell while the permeate side is evacuated, then the feed side is evacuated too. After that the valve on the feed side is closed and put under defined pressure (e.g., 3 bars) with a single gas (beginning with the slower permeating gas, *i.e.*, nitrogen if oxygen/nitrogen separation is investigated). After pressurizing the feed side for 2 h the permeation measurements can be started. The line between the permeate side and the vacuum pump is closed and the feed pressure adjusted. Since the gas permeates from the feed side through the membrane to the permeate side the pressure there increases. The linear pressure rise is recorded with an *x*–*y* printer and used to calculate the permeability *P* in barrer units (1 barrer = 1 × 10^−10^ cm^3^ (STP) cm/cm^2^ s cmHg or, in SI units, 7.5005 × 10^−18^ m^2^·s^−1^·Pa^−1^, see Supplementary Information) (Equation 1).

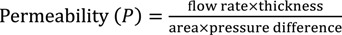
(1)


From the single gas permeabilities the ideal gas selectivity was calculated according to the Equation (2):

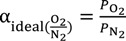
(2)


For the pure polymer, four individual membranes with an area of 11.3 cm^2^ and thicknesses of about 30 µm were analyzed in gas permeation experiments. In the case of the MMMs, samples with an area of around 11.3 cm^2^ and thicknesses between 30 and 75 µm were analyzed. Each gas permeation was measured three times with the same membrane (pure polymer and MMMs) for error estimation.

### 2.6. Positron Annihilation Lifetime Spectroscopy (PALS)

#### 2.6.1. Measurements

**Figure 4:**
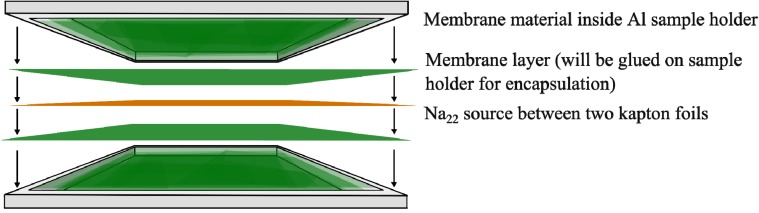
Schematic presentation of sample preparation for positron annihilation lifetime spectroscopy (PALS).

Positron annihilation experiments have been performed in a fast-fast coincidence setup with a homemade temperature-controllable sample holder under high vacuum conditions as described, e.g., in reference [89]. Experiments were performed with two different time resolutions and sources for mixed-matrix membranes and pure MOFs to take into account the long lifetimes in the MOF. For the measurements the membranes were cut into small pieces and filled into small aluminum pans (Figure 4). The pure MIL-101 was also filled into aluminum pans. Pans were stacked with a Na-22 source (1 MBq, for mixed-matrix membranes and 0.1 MBq for pure MOF, both encapsulated in Kapton) in a sandwich like manner (total thickness ≈ 1.2 mm) to ensure complete absorption of the positrons in the sample. The pans were sealed with another membrane layer on the top and the whole device mounted into a sample holder in vacuum (10^−5^ mbar). Special care was taken to ensure relative comparison, *i.e.*, same Na-22 source, same counting rate, same temperature program, same evaluation method and boundary conditions were used for all mixed-matrix membrane samples, only the pure MOF was analyzed separately (see discussion).

Positron annihilation spectra were recorded at a counting rate of *ca.* 350 cts/s with a total of typically 5 × 10^6^ cts within several hours. The temperature was set to 30 °C, accuracy ±1 K. Evaluation was performed with the LT9.2 routine program [90], using the common background subtraction and the final resolution function, which was determined as a sum of two Gaussians with FWHMs (Full width at half maximum) of approx. 268 ps and 445 ps and weight of 80% and 20%; respectively. Up to five lifetime components were assumed. The resolution function was fixed for all spectra. Also τ_1_ was fixed at 125 ps for MMMs because further lifetime components, related to annihilation in the MOF were introduced. The fit of all other parameters was free.

#### 2.6.2. Conversion o-Ps Lifetime to Hole Size (Tao-Eldrup Model)

Once injected from a radioactive source, positrons form in most polymers hydrogen-like positronium (Ps) states. The pick-off lifetime of ortho-positronium (τ_o-Ps_), is well correlated to the free-volume hole size in polymers. The success of PALS in polymer research is largely due to the so-called standard model developed by Tao and Eldrup [78], later extended to larger holes [91]. This simple quantum mechanical model assumes the Ps to be confined to spherical holes with infinitely high walls and gives a direct relationship between τ_o-Ps_ and the size of the free volume holes. This can quantitatively be expressed as Equation (3):

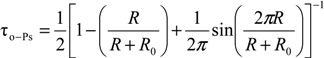
(3)
where *R* is the average radius of holes (free volume element), *R*_0_* =* *R +* *∆R* and *∆R* = 0.166 nm [92]. 

The interpretation of the o-Ps intensity, which has often been used as a measure for the hole concentration in polymers, is questionable, as the intensity is also affected by the positronium formation probability [93]. However, for binary mixtures (here polysulfone and MOF), the intensity of the respective components can be correlated to their respective concentrations of polymer and MOF in the sample. 

## 3. Results and Discussion

### 3.1. Gas Permeation

**Figure 5:**
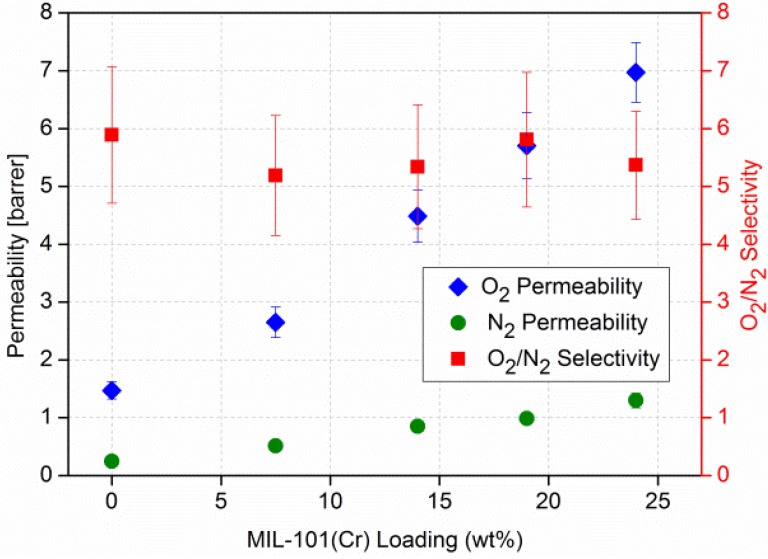
O_2_/N_2_ permeability and separation performance of pure polysulfone (PSF) and MIL-101/PSF membranes with different MIL wt % loadings (graphics with revised MIL-101 wt % [94] values compared to reference [85]).

**Figure 6:**
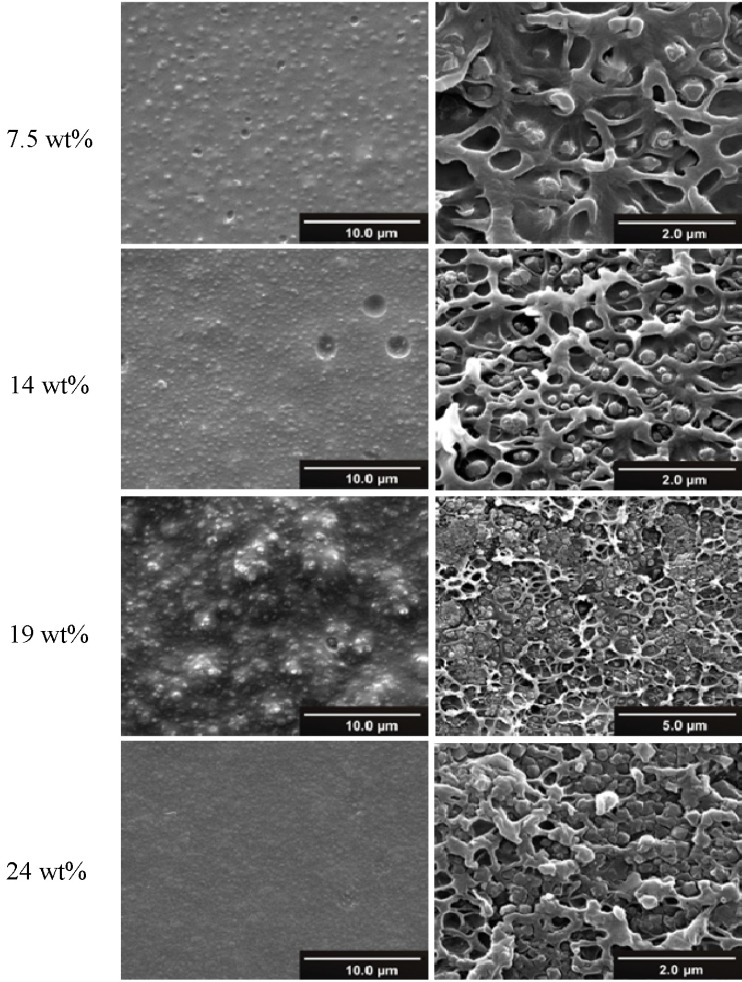
SEM photographs of MIL-101/PSF membranes based on 400 mg of PSF with different loadings of MIL-101. Left: membranes surface; right: cross section view.

We had communicated the results of a study of polysulfone (PSF) mixed-matrix membranes containing the water-stable MIL-101(Cr). These MIL-101/PSF MMMs exhibited a remarkable four-fold increase in the permeability of O_2_ to technically needed values above 6 barrer, thereby, keeping the high PSF selectivity for O_2_ over N_2_ of 5–6 (Figure 5). High loads up to 24 wt % [94] of MIL-101(Cr) in PSF could be achieved with the MIL-101 particles showing very good adhesion with polysulfone in the mixed-matrix membranes (Figure 6) and excellent long term stability. Particle aggregation becomes obvious at the 19% loading (Figure 6c,d). The MMMs remain flexible even at 19% loading and only start to markedly lose their flexibility at 24% loading. A comparison of these results with the separation performance of other MOF-MMMs shows that the MIL-101/PSF membranes exhibit much higher O_2_ permeabilities (>4 barrer) than any other MOF-based mixed-matrix membranes [15,85]. MIL-101/PSF was just recently surpassed for O_2_/N_2_ separation by the higher gas permeation performance of ZIF-8/PIM-1 nanocomposite membranes. In fact ZIF-8/PIM-1 performed above the 2008 upper bound for several gas pairs [84].

The polysulfone polymer does not appear impressive in terms of its separation performance. However, PSF is employed on the scale of several thousand tons per year for membranes for dialysis and water treatment. Polysulfone is one of the most important glassy polymers used in industrial membrane gas separation [6,95]. PSF is characterized through high temperature stability and very good mechanical properties. Further, it is industrially available with high molecular weights and consistent specifications so that deviations which are observed from different polymer batches or impurities can therefore be eliminated. 

**Figure 7:**
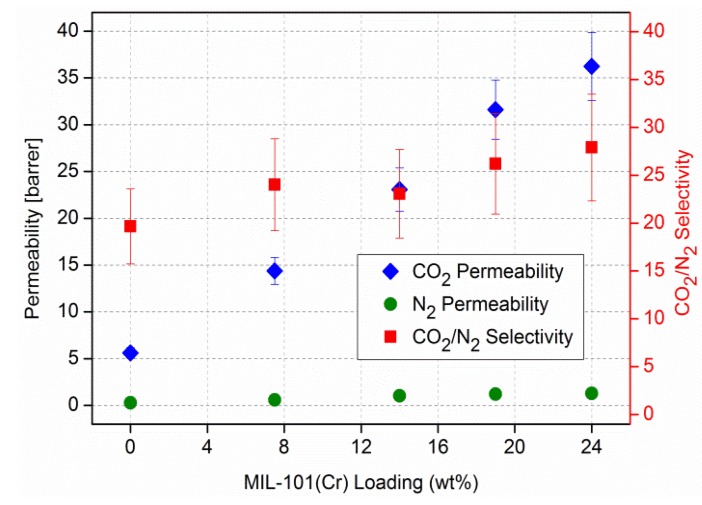
CO_2_/N_2_ permeability and separation performance of pure PSF and MIL-101/PSF membranes with different MIL wt % loadings.

**Figure 8:**
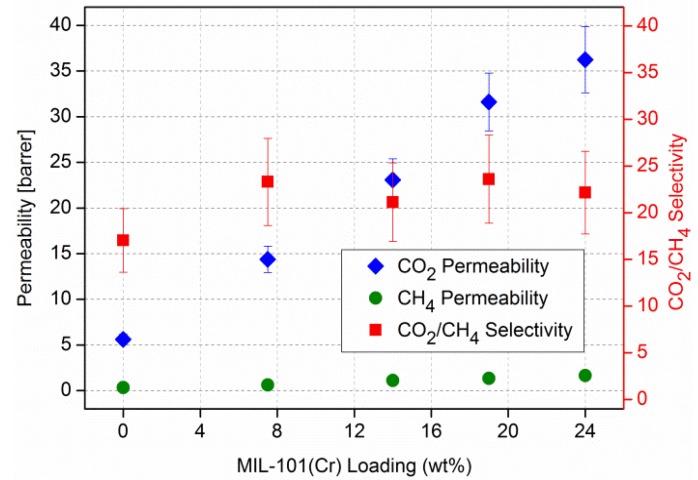
CO_2_/CH_4_ permeability and separation performance of pure PSF and MIL-101/PSF membranes with different MIL wt % loadings.

Single gas experiments with CO_2_, CH_4_ and N_2_ on the MIL-101/PSF membranes at different MOF loadings yielded increases in gas permeabilities with increasing MIL-101 weight percentage in PSF (Figure 7 and Figure 8) [96]. CO_2_ is the faster permeating gas. The CO_2_ permeability increases from about 5 to over 35 barrer from pure PSF to 24 wt % MIL-101/PSF. The increase for CO_2_ also raises the ideal selectivities for CO_2_/N_2_ and CO_2_/CH_4_ from about 20 to 25 [96]. 

**Figure 9:**
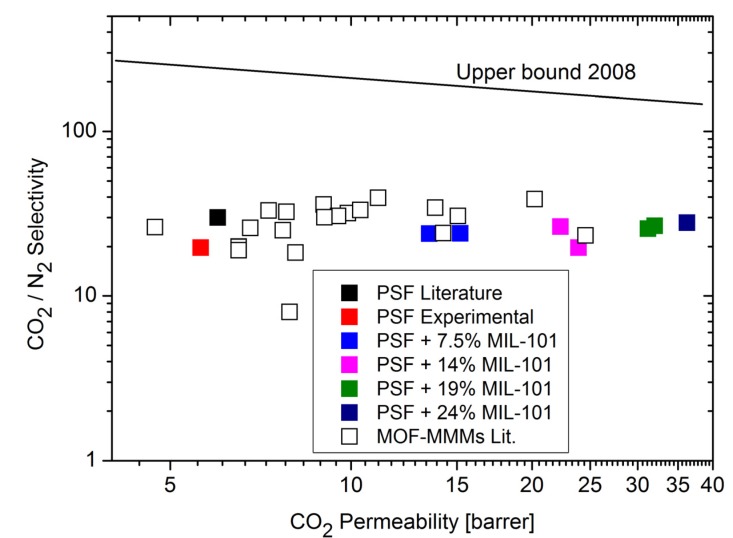
Comparison of CO_2_/N_2_ separation performance of MIL-101/PSF with other metal-organic framework (MOF)-containing mixed-matrix membranes from literature data (for further details on MOF-mixed-matrix membrane (MMM) data points see Table S2 and Table S4, Figure S6). The Robeson upper bound for polymer separation performance as defined 2008 is shown [10].

A comparison of the CO_2_/N_2_ results with the separation performance of other MOF-MMMs shows that the MIL-101/PSF membranes exhibit again higher CO_2_ permeabilities at its highest loading (>30 barrer) than most other MOF-based mixed-matrix membranes (Figure 9). ZIF-8/PIM-1 membranes showed CO_2_ permeabilities of about 4000–6000 barrer [84].

**Figure 10:**
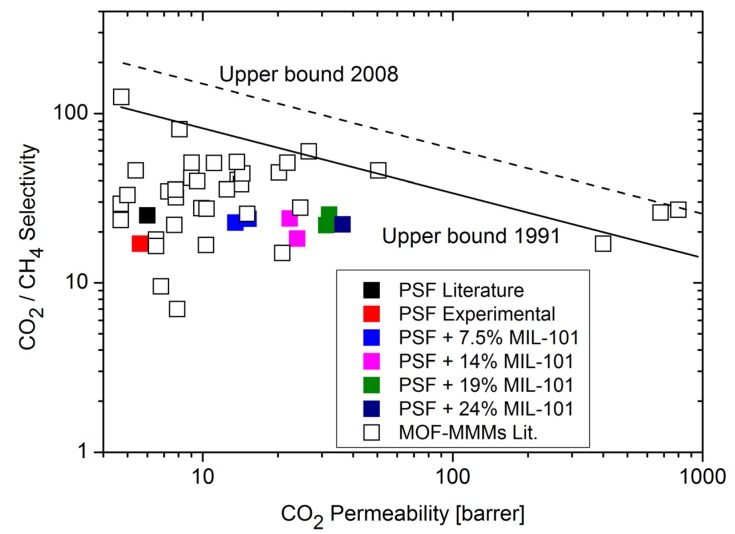
Comparison of CO_2_/CH_4_ separation performance of MIL-101/PSF with other MOF-containing mixed-matrix membranes from literature data (for further details on MOF-MMM data points see Table S3 and Table S5, Figure S7). The Robeson upper bounds for polymer separation performance as defined in 1991 and 2008 are shown [9,10].

For the CO_2_/CH_4_ separation the MIL-101/PSF membranes performed well but cannot surpass membranes made from ZIF-90/6FDA–DAM [6FDA: 2,2-bis(3,4-carboxyphenyl) hexafluoro-propane dianhydride; DAM: diaminomesitylene] [27] (Figure 10) and that of PIM-1/ZIF-8 [84]. The permeation properties of both pure highly permeable polymer 6FDA–DAM and 15 wt % ZIF-90/6FDA–DAM membranes were investigated for a CO_2_/CH_4_ gas mixture and show an enhancement of gas-separation performance of the ZIF-90/6FDA–DAM membranes [27]. Also an increase in the content of nano ZIF-8 (up to 43 wt %) in ZIF-8/PIM-1 MMMs increased the permeability coefficients [84].

The experimental permeability of gas species through MMMs can be compared with theoretical predictions by several models [97]. The Maxwell and Bruggeman permeation models are usually used to assess the permeabilities of gases through MMMs [11,12,98]. The Maxwell model describes the effective permeability (*P*_eff_) of gas species for a dispersion of filler particles in a polymer matrix, that is, a mixed-matrix membrane. The Maxwell equation can be expressed by Equation (4):

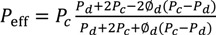
(4)


*P_c_* is the permeability of the continuous (pure) polymer phase, and *P_d_* is the permeability of the dispersed phase. Φ*_d_* is the volume fraction of the dispersed phase as given in Equation (5): 

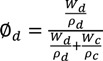
(5)


*W_d_* and *W_c_* are the weight and *ρ_d_* and *ρ_c_* the density of the dispersed filler and continuous polymer, respectively. The densities may be taken from the literature or determined experimentally.

By defining a “reduced permeation polarizability” β as (Equation (6)) [11],

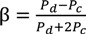
(6)


Equation (4) can be rewritten to Equation (7):

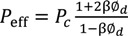
(7)


The value of β is a measure of the permeability difference between the polymer phase (with *P_c_*) and the dispersed MOF phase (with *P_d_*). Three limiting cases exist: For highly permeable MOFs *P_d_* >> *P_c_* and β ≈ 1; for equal permeability in both phases β ≈ 0 and for non-permeable filler (*P_d_* = 0) β ≈ −0.5 [11]. 

The Maxwell model is intended to be applicable for low filler loadings (Φ*_d_* < 0.2) since it assumes that the streamlines associated with diffusive mass transport around filler particles are not affected by the presence of nearby particles [99]. The Bruggeman model is an improved version of the Maxwell model [100], can be used at higher loads and correlates the effective permeability (*P*_eff_) with the volume fraction Φ*_d_* of the dispersed phase in Equation (8):

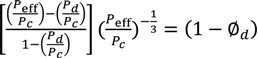
(8)


For the maximum and minimum limits, the ratios of the effective permeability of the MMM (*P_eff_*) relative to the continuous (pure) polymer matrix (*P_c_*) permeability are given in Equation (9) [99]:

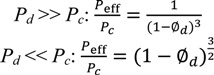
(9)


The above Maxwell and Bruggeman models give similar results up to Φ*_d_* ≈ 0.2 [99]. 

**Figure 11:**
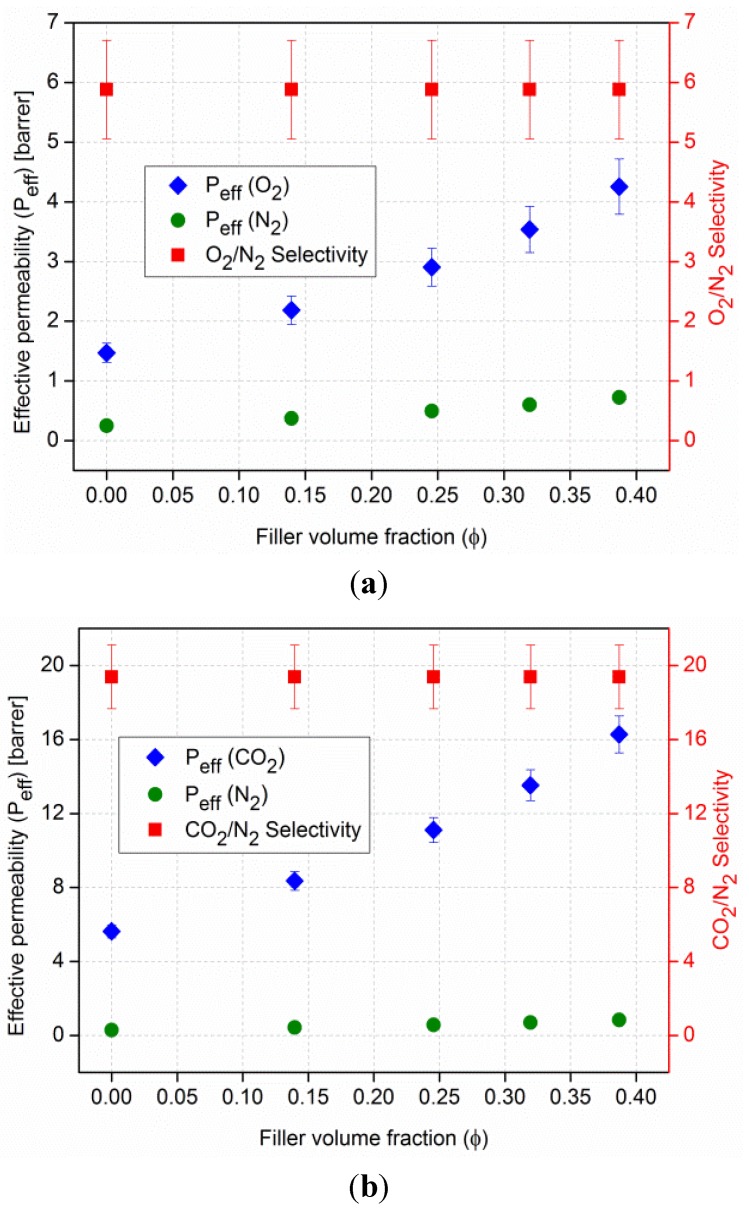
Calculated permeability and selectivity for the MIL-101/PSF-MMMs using the Maxwell model under the assumption of a highly permeable dispersed (filler, MOF) phase, *i.e.*, *P_d_* >> *P_c_* and β ≈ 1. Part (**a**) is to be compared with Figure 5 and (**b**) with Figure 7.

Use of the Maxwell model with β ≈ 1 reproduces the approximate effective permeabilities and the ideal selectivity of ~6 and ~20 for the O_2_/N_2_ and CO_2_/N_2_ separation performance of the MIL-101/PSF membranes with different filler wt % loadings (given here filler volume fractions Φ*_d_*) (Figure 11). Figure 11 is to be compared with Figure 5 and Figure 7, respectively. Filler volume fractions of 0.14, 0.25, 0.32 and 0.39 correspond to MIL-101 wt % loadings of 7.5, 14, 19 and 24 wt %, respectively (see Table S1 and Figure S5). We assign the lower calculated CO_2_ permeability to the limiting assumptions of the Maxwell model. 

### 3.2. PALS Experiments

In order to attribute the o-Ps lifetimes reasonably to the respective contributions in the mixed-matrix membrane samples, first the pure constituents are discussed. For the pure polysulfone only one o-Ps lifetime of 2.1 ns and an intensity *I*_3_ of 20% is found and both values are typical for polymers (Table S6 and Table S7) [93]. 

For the pure porous MIL-101 material PALS will probe an inner free volume with a diameter of ~1.2 nm for pentagonal windows or ~1.47 nm for hexagonal windows but not ~3 nm for the mesoporous cages (*cf.* Figure 2) as PALS does only probe the shortest diameter [101,102]. Table 1 lists the diameters, the radii subtracted with the van der Waals radii of 0.14 nm and the expected o-Ps lifetimes due to the Tao-Eldrup model (see Section 2.6). As already indicated in the literature [103], there are longer lifetime contributions (~80 ns) in the spectra, which might arise from delocalized positronium states and, thus, represent an “artefact” of the measurement. We evaluated the corresponding spectra for the pure MOF with five and six components, the corresponding results are summarized in Table S6 and Table S7. 

**Table 1:** Relevant distances and ortho-positronium lifetimes in MIL-101 for PALS.

Inner free volume ^a^	Diameter (nm) ^a^	Effective radius (nm) ^b^	Expected o-Ps lifetime (ns) ^c^
Pentagonal window	1.2	0.46	4.7
Hexagonal window	1.47	0.595	8
small meso-cage 1	2.9	1.31	30.5
large meso-cage 2	3.4	1.56	38

^a^ see Figure 2; ^b^ effective radius = diameter/2 − 0.14 nm (vdW radius); ^c^ o-Ps = ortho-positronium.

**Figure 12:**
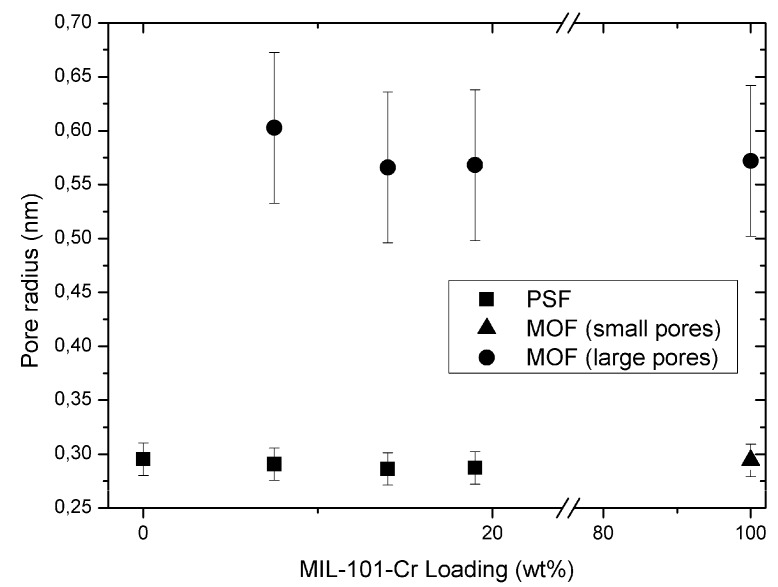
Pore radii of the average free volume as function of wt % MIL-101 in MMM. For details see Section 2.6.2.

**Figure 13:**
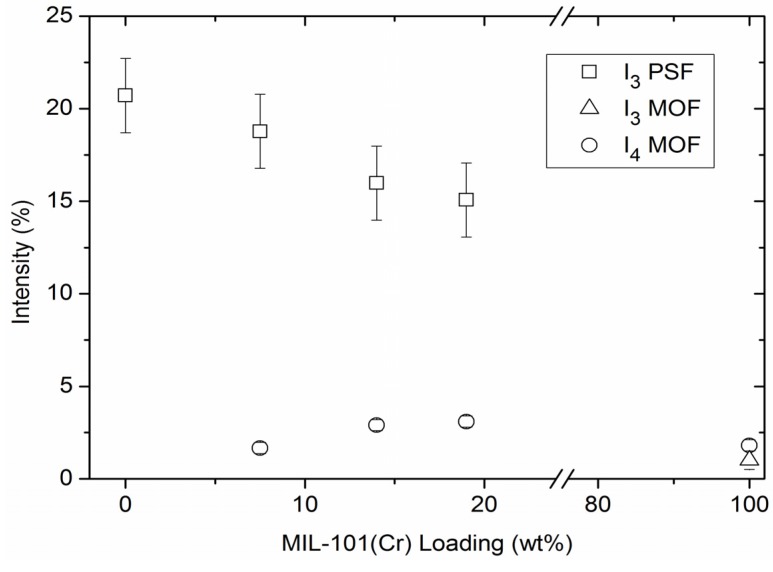
o-Ps intensities as function of wt % MIL-101 in MMM. The o-Ps intensity is related to the relative contribution of annihilation events in the respective phase of the sample. For details see Section 2.6.2.

Figure 12 displays the average free volume radii versus the weight content of MIL-101 in PSF. In Figure 13 the respective intensities are plotted *vs*. MOF content. Specific values are listed in Table S6 and Table S7.

From Figure 12 it is obvious that there is no systematic change in the free volume radii of the PSF polymer and the MOF filler with different weight fractions. Neither the free volume radius of the polymer nor the pore radius of MIL-101 is altered by combining both in a mixed-matrix membrane with increasing MIL-101 content. Hence, the structure of the MIL-101 particles seems to be unaffected by the polymer and *vice versa*. No additional free volume is introduced in the polymer or at the MOF-polymer interface with the addition of the MIL-101 filler. Instead free volume sites inside the MIL-101 particles were brought into the MMM. Furthermore, one can assume that the mesocages in MIL-101 are not filled by polymer material. The mean value of the o-Ps lifetime in the MOF filler is 7.8 ns, and the values of Table 1 indicate clearly that the o-Ps probes the pores of MIL-101.

In Figure 13 the intensity of the third PSF-component decreases nearly linear with MIL-101 concentration and the fourth MOF-component increases with MIL-101 concentration. This can be explained easily, as with less (more) concentration of a component there will be less (more) contribution of the respective lifetime of that component. In other words, the intensity *I*_3_ of the third lifetime component τ_3_ which is associated with PSF decreases with the decreasing polymer content at the expense of increasing MIL-101 filler weight fraction. The intensity *I*_4_ of the fourth lifetime component τ_4_ is associated with MIL-101 filler content and increases accordingly. This graph is still a good proof for the statement that the third component can be contributed to a mixed lifetime of PSF and MIL-101 and the fourth component only to the MIL-101.

## 4. Conclusions

Isochorus, that is, single gas CO_2_, N_2_ and CH_4_ permeation experiments on MIL-101/PSF membranes show an almost linear increase in permeability for the fast gas CO_2_ with slight increases in ideal CO_2_/N_2_ and CO_2_/CH_4_ selectivities with increasing MIL-101 content. The CO_2_/N_2_ separation performance of MIL-101/PSF with 19 wt % filler content surpasses those of most other known MOF-MMMs. The Maxwell model is able to reproduce the approximate effective permeabilities and the ideal selectivity for the O_2_/N_2_ and CO_2_/N_2_ separation performance of the MIL-101(Cr)/PSF membranes with different filler loadings under the assumption of a higher gas permeability *P* in dispersed MOF phase than in the continuous polymer phase, that is, *P_d_* >> *P_c_*.

In PALS experiments the MIL-101/PSF-MMM samples show only minor changes in the lifetime of the third and fourth component, τ_3_ and τ_4_ with increasing wt % of MIL-101. But the MMM samples show a decreasing intensity for the third lifetime component and an increasing intensity for the fourth lifetime component paralleling the increased MIL-101 filler content. This indicates that the increased permeability for N_2_ and O_2_ in the MIL-101/PSF-MMMs is not due to free volume changes in the PSF but due to the added large free volume inside the MIL-101 filler particles.

## Supplementary Files

Supplementary File 1 Supplementary Information (PDF, 1378 KB)

## References

[1] Davis J.C., Valus R.J., Eshraghi R., Velikoff A.E. (1993). Facilitated transport membrane hybrid systems for olefin purification. Sep. Sci. Technol..

[2] Strathmann H. (2001). Membrane separation processes: Current relevance and future opportunities. AIChE J..

[3] He X., Hägg M.-J. (2012). Membranes for environmentally friendly energy processes. Membranes.

[4] Koros W.J., Mahajan R. (2000). Pushing the limits on possibilities for large scale gas separation: Which strategies?. J. Membr. Sci..

[5] Ohlrogge K., Stürken K. (2001). The Separation of Organic Vapors from Gas Streams by Means of Membranes. Membrane Technology.

[6] Baker R.W. (2002). Future directions of membrane gas separation technology. Ind. Eng. Chem. Res..

[7] BORSIG Membrane Technology GmbH. http://www.borsig-china.com/#productrecovery.

[8] Sulzer Chemtech AG. http://www.sulzerchemtech.com.

[9] Robeson L.M. (1991). Correlation of separation factor versus permeability for polymeric membranes. J. Membr. Sci..

[10] Robeson L.M. (2008). The upper bound revisited. J. Membr. Sci..

[11] Basu S., Cano-Odena A., Vankelecom I.F.J. (2010). Asymmetric Matrimid^®^/[Cu_3_(BTC)_2_] mixed-matrix membranes for gas separations. J. Membr. Sci..

[12] Chung T.S., Jiang L.Y., Li Y., Kulprathipanja S. (2007). Mixed matrix membranes (MMMs) comprising organic polymers with dispersed inorganic fillers for gas separation. Prog. Polym. Sci..

[13] Zornoza B., Martinez-Joaristi A., Serra-Crespo P., Tellez C., Coronas J., Gascon J., Kapteijn F. (2011). Functionalized flexible MOFs as fillers in mixed matrix membranes for highly selective separation of CO_2_ from CH_4_ at elevated pressures. Chem. Commun..

[14] Nik O.G., Chen X.Y., Kaliaguine S. (2012). Functionalized metal organic framework-polyimide mixed matrix membranes for CO_2_/CH_4_ separation. J. Membr. Sci..

[15] Tanh Jeazet H.B., Staudt C., Janiak C. (2012). Metal-organic frameworks in mixed-matrix membranes for gas separation. Dalton Trans..

[16] Zornoza B., Tellez C., Coronas J., Gascon J., Kapteijn F. (2013). Metal organic framework based mixed matrix membranes: An increasingly important field of research with a large application potential. Microporous Mesoporous Mater..

[17] Bastani D., Esmaeili N., Asadollahi M. (2013). Polymeric mixed matrix membranes containing zeolites as a filler for gas separation applications: A review. J. Ind. Eng. Chem..

[18] Dong G., Li H., Chen V. (2013). Challenges and opportunities for mixed-matrix membranes for gas separation. J. Mater. Chem. A.

[19] Ordonez M.J.C., Balkus K.J., Ferraris J.P., Musselman I.H. (2010). Molecular sieving realized with ZIF-8/Matrimid (R) mixed-matrix membranes. J. Membr. Sci..

[20] Dai Y., Johnson J.R., Karvan O., Sholl D.S., Koros W.J. (2012). Ultem/ZIF-8 mixed matrix hollow fiber membranes for CO_2_/N_2_ separations. J. Membr. Sci..

[21] Rebollar-Perez G., Carretier E., Lesage N., Moulin P. (2011). Volatile organic compound (VOC) removal by vapor permeation at low VOC concentrations: Laboratory scale results and modeling for scale up. Membranes.

[22] Dumee L., Velleman L., Sears K., Hill M., Schutz J., Finn N., Duke M., Gray S. (2011). Control of porosity and pore size of metal reinforced carbon nanotube membranes. Membranes.

[23] Noble R.D. (2011). Perspectives on mixed matrix membranes. J. Membr. Sci..

[24] Li J.R., Ma Y.G., McCarthy M.C., Sculley J., Yu J.M., Jeong H.K., Balbuena P.B., Zhou H.C. (2011). Carbon dioxide capture-related gas adsorption and separation in metal-organic frameworks. Coord. Chem. Rev..

[25] Liu D.H., Zhong C.L. (2010). Understanding gas separation in metal-organic frameworks using computer modeling. J. Mater. Chem..

[26] Meek S.T., Greathouse J.A., Allendorf M.D. (2011). Metal-organic frameworks: A rapidly growing class of versatile nanoporous materials. Adv. Mater..

[27] Bae T.H., Lee J.S., Qiu W.L., Koros W.J., Jones C.W., Nair S. (2010). A high-performance gas-separation membrane containing submicrometer-sized metal-organic framework crystals. Angew. Chem. Int. Ed..

[28] Kitagawa S., Matsuda R. (2007). Chemistry of coordination space of porous coordination polymers. Coord. Chem. Rev..

[29] Maji T.K., Kitagawa S. (2007). Chemistry of porous coordination polymers. Pure Appl. Chem..

[30] Janiak C. (2003). Engineering coordination polymers towards applications. Dalton Trans..

[31] Janiak C., Vieth J.K. (2010). MOFs, MILs and more: Concepts, properties and applications for porous coordination networks (PCNs). New J. Chem..

[32] Long J.R., Yaghi O.M. (2009). The pervasive chemistry of metal-organic frameworks. Chem. Soc. Rev..

[33] Biradha K. (2010). Introduction to the themed issue “Coordination polymers: Structure and function”. New J. Chem..

[34] Zaworotko M.J. (2010). There is plenty of room in the middle: Crystal clear opportunities abound for coordination polymers. New J. Chem..

[35] Kitagawa S., Natarajan S. (2010). Targeted fabrication of MOFs for hybrid functionality. Eur. J. Inorg. Chem..

[36] Zhou H.C., Long J.R., Yaghi O.M. (2012). Introduction to metal-organic frameworks. Chem. Rev..

[37] Czaja A.U., Trukhan N., Müller U. (2009). Industrial applications of metal-organic frameworks. Chem. Soc. Rev..

[38] Férey G. (2009). Some suggested perspectives for multifunctional hybrid porous solids. Dalton Trans..

[39] Prakash M.J., Lah M.S. (2009). Metal-organic macrocycles, metal-organic polyhedra and metal-organic frameworks. Chem. Commun..

[40] Wu H., Gong Q., Olson D.H., Li J. (2012). Commensurate adsorption of hydrocarbons and alcohols in microporous metal organic frameworks. Chem. Rev..

[41] Li K., Olson D.H., Li J. (2010). Commensurate adsorption of hydrocarbons in microporous metal-organic frameworks. Trends Inorg. Chem..

[42] Murray L.J., Dinca M., Long J.R. (2009). Hydrogen storage in metal-organic frameworks. Chem. Soc. Rev..

[43] Li J.-R., Kuppler R.J., Zhou H.-C. (2009). Selective gas adsorption and separation in metal-organic frameworks. Chem. Soc. Rev..

[44] Morris R.E., Wheatley P.S. (2008). Gas storage in nanoporous materials. Angew. Chem. Int. Ed..

[45] Paik Suh M., Park H.J., Prasad T.K., Lim D.-W. (2012). Hydrogen storage in metal-organic frameworks. Chem. Rev..

[46] Paik Suh M., Cheon Y.E., Lee E.Y. (2008). Syntheses and functions of porous metallosupramolecular networks. Coord. Chem. Rev..

[47] Düren T., Bae Y.-S., Snurr R.Q. (2009). Using molecular simulation to characterise metal-organic frameworks for adsorption applications. Chem. Soc. Rev..

[48] Han S.S., Mendoza-Cortés J.L., Goddard W.A. (2009). Recent advances on simulation and theory ofhydrogen storage in metal-organic frameworks and covalent organic frameworks. Chem. Soc. Rev..

[49] Getman R.B., Bae Y.-S., Wilmer C.E., Snurr R.Q. (2012). Review and analysis of molecular simulations of methane, hydrogen, and acetylene storage in metal-organic frameworks. Chem. Rev..

[50] Chen Z., Xiang S., Arman H.D., Li P., Tidrow S., Zhao D., Chen B. (2010). A microporous metal-organic framework with immobilized –OH functional groups within the pore surfaces for selective gas sorption. Eur. J. Inorg. Chem..

[51] Ma F., Liu S., Liang D., Ren G., Zhang C., Wei F., Su Z. (2010). Hydrogen adsorption in polyoxometalate hybrid compounds based on porous metal-organic frameworks. Eur. J. Inorg. Chem..

[52] Kepert C.J. (2006). Advanced functional properties in nanoporous coordination framework materials. Chem. Commun..

[53] Zhang Z., Zhao Y., Gong Q., Li Z., Li J. (2013). MOFs for CO_2_ capture and separation from flue gas mixtures: The effect of multifunctional sites on their adsorption capacity and selectivity. Chem. Commun..

[54] Li J.-R., Sculley J., Zhou H.-C. (2012). Metal-organic frameworks for separations. Chem. Rev..

[55] Hao G.-P., Li W.-C., Lu A.-H. (2011). Novel porous solids for carbon dioxide capture. J. Mater. Chem..

[56] Férey G., Serre C., Devic T., Maurin G., Jobic H., Llewellyn P.L., de Weireld G., Vimont A., Daturi M., Chang J.-S. (2011). Why hybrid porous solids capture greenhouse gases?. Chem. Soc. Rev..

[57] Nune S.K., Thallapally P.K., McGrail B.P. (2010). Metal organic gels (MOGs): A new class of sorbents for CO2 separation applications. J. Mater. Chem..

[58] Cychosz K.A., Ahmad R., Matzger A.J. (2010). Liquid phase separations by crystalline microporous coordination polymers. Chem. Sci..

[59] Horcajada P., Gref R., Baati T., Allan P.K., Maurin G., Couvreur P., Férey G., Morris R.E., Serre C. (2012). Metal-organic frameworks in biomedicine. Chem. Rev..

[60] Lohe M.R., Gedrich K., Freudenberg T., Kockrick E., Dellmann T., Kaskel S. (2011). Heating and separation using nanomagnet-functionalized metal-organic frameworks. Chem. Commun..

[61] Férey G. (2008). Hybrid porous solids: Past, present, future. Chem. Soc. Rev..

[62] Bétard A., Fischer R.A. (2012). Metal-organic framework thin films: From fundamentals to applications. Chem. Rev..

[63] Takashima Y., Martínez V.M., Furukawa S., Kondo M., Shimomura S., Uehara H., Nakahama M., Sugimoto K., Kitagawa S. (2011). Molecular decoding using luminescence from an entangled porous framework. Nat. Commun..

[64] Halder G.J., Kepert C.J., Moubaraki B., Murray K.S., Cashion J.D. (2002). Guest-dependent spin crossover in a nanoporous molecular framework material. Science.

[65] Yoon M., Srirambalaji R., Kim K. (2012). Homochiral metal-organic frameworks for asymmetric heterogeneous catalysis. Chem. Rev..

[66] Ladrak T., Smulders S., Roubeau O., Teat S.J., Gamez P., Reedijk J. (2010). Manganese-based metal-organic frameworks as heterogeneous catalysts for the cyanosilylation of acetaldehyde. Eur. J. Inorg. Chem..

[67] Kleist W., Jutz F., Maciejewski M., Baiker A. (2009). Mixed-linker metal-organic frameworks as catalysts for the synthesis of propylene carbonate from propylene oxide and CO_2_. Eur. J. Inorg. Chem..

[68] Ma L., Abney C., Lin W. (2009). Enantioselective catalysis with homochiral metal-organic frameworks. Chem. Soc. Rev..

[69] Lee J., Farha O.K., Roberts J., Scheidt K.A., Nguyen S.T., Hupp J.T. (2009). Metal-organic framework materials as catalysts. Chem. Soc. Rev..

[70] Farrusseng D., Aguado S., Pinel C. (2009). Metal-organic frameworks: Opportunities for catalysis. Angew. Chem. Int. Ed..

[71] Meilikhov M., Yusenko K., Esken D., Turner S., van Tendeloo G., Fischer R.A. (2010). Metals@MOFs—Loading MOFs with metal nanoparticles for hybrid functions. Eur. J. Inorg. Chem..

[72] Falcaro P., Hill A.J., Nairn K.M., Jasieniak J., Mardel J.I., Bastow T.J., Mayo S.C., Gimona M., Gomez D., Whitfield H.J. (2011). A new method to position and functionalize metal-organic framework crystals. Nat. Commun..

[73] Khutia A., Rammelberg H.U., Schmidt T., Henninger S.K., Janiak C. (2013). Water sorption cycle measurements on functionalized MIL-101Cr for heat transformation application. Chem. Mater..

[74] Henninger S.K., Jeremias F., Kummer H., Janiak C. (2012). MOFs for use in adsorption heat pump processes. Eur. J. Inorg. Chem..

[75] Jeremias F., Khutia A., Henninger S.K., Janiak C. (2012). MIL-100(Al, Fe) as water adsorbents for heat transformation purposes—A promising application. J. Mater. Chem..

[76] Ehrenmann J., Henninger S.K., Janiak C. (2011). Water adsorption characteristics of MIL-101 for heat-transformation applications of MOFs. Eur. J. Inorg. Chem..

[77] Henninger S.K., Habib H.A., Janiak C. (2009). MOFs as adsorbents for low temperature heating and cooling applications. J. Am. Chem. Soc..

[78] Tao S.J. (1972). Positronium annihilation in molecular substances. J. Chem. Phys..

[79] Eldrup M., Lightbody D., Sherwood J.N. (1981). The temperature-dependence of positron lifetimes in solid pivalic acid. Chem. Phys..

[80] Kruse J., Rätzke K., Faupel F., Sterescu D.M., Stamatialis D.F., Wessling M. (2007). Free volume in C-60 modified PPO polymer membranes by positron annihilation lifetime spectroscopy. J. Phys. Chem. B.

[81] Konietzny R., Barth C., Harms S., Rätzke K., Kölsch P., Staudt C. (2011). Structural investigations and swelling behavior of 6FDA copolyimide thin films. Polym. Int..

[82] Harms S., Rätzke K., Zaporojtchenko V., Faupel F., Egger W., Ravelli L. (2011). Free volume distribution at the Teflon AF (R)/silicon interfaces probed by a slow positron beam. Polymer.

[83] Song Q., Nataraj S.K., Roussenova M.V., Tan J.C., Hughes D.J., Li W., Bourgoin P., Alam M.A., Cheetham A.K., Al-Muhtaseb S.A. (2012). Zeolitic imidazolate framework (ZIF-8) based polymer nanocomposite membranes for gas separation. Energy Environ. Sci..

[84] Bushell A.F., Attfield M.P., Mason C.R., Budd P.M., Yampolskii Y., Starannikova L., Rebrov A., Bazzarelli F., Bernardo P., Jansen J.C. (2013). Gas permeation parameters of mixed matrix membranes based on the polymer of intrinsic microporosity PIM-1 and the zeolitic imidazolate framework ZIF-8. J. Membr. Sci..

[85] Tanh Jeazet H.B., Staudt C., Janiak C. (2012). A method for increasing permeability in O_2_/N_2_ separation with mixed-matrix membranes made of water-stable MIL-101 and polysulfone. Chem. Commun..

[86] Férey G., Mellot-Draznieks C., Serre C., Millange F., Dutour J., Surble S., Margiolaki I. (2005). A chromium terephthalate-based solid with unusually large pore volumes and surface area. Science.

[87] Brandenburg K. (2007–2012). Diamond—Crystal and Molecular Structure Visualization.

[88] Wieneke J.U., Staudt C. (2010). Thermal stability of 6FDA-(co-)polyimides containing carboxylic acid groups. Polym. Degrad. Stab..

[89] Kruse J., Kanzow J., Rätzke K., Faupel F., Heuchel M., Frahn J., Hofmann D. (2005). Free volume in polyimides: Positron annihilation experiments and molecular modeling. Macromolecules.

[90] Kansy J. (1996). Microcomputer program for analysis of positron annihilation lifetime spectra. Nucl. Instrum. Methods A.

[91] Dull T.L., Frieze W.E., Gidley D.W., Sun J.N., Yee A.F. (2001). Determination of pore size in mesoporous thin films from the annihilation lifetime of positronium. J. Phys. Chem. B.

[92] Nagel C., Schmidtke E., Günther-Schade K., Hofmann D., Fritsch D., Strunskus T., Faupel F. (2000). Free volume distributions in glassy polymer membranes: Comparison between molecular modeling and experiments. Macromolecules.

[93] Jean Y.C., Mallon P.E., Schrader D.M. (2003). Principles and Applications of Positron & Positronium Chemistry.

[B94-membranes-03-00331] 94.MIL-101 mass fractions in the MMM had been erroneously calculated somewhat too high in reference [85]. Instead of 8, 16 and 24 wt % the correct mass fractions are 7.5, 14 and 19 wt %, respectively for MIL-101 in PSF. This recalculation does not change the conclusions drawn in reference [85]. To the contrary, the increase in permeability is already achieved with even lower MIL-101 mass fractions.

[95] Bernardo P., Drioli E., Golemme G. (2009). Membrane gas separation: A review/state of the art. Ind. Eng. Chem. Res..

[96] Hunger K., Schmeling N., Tanh Jeazet H.B., Janiak C., Staudt C., Kleinermanns K. (2012). Investigation of cross-linked and additive containing polymer materials for membranes with improved performance in pervaporation and gas separation. Membranes.

[97] Petropoulos J.H. (1985). A comparative-study of approaches applied to the permeability of binary composite polymeric materials. J. Polym. Sci..

[98] Keskin S., Sholl D.S. (2010). Selecting metal organic frameworks as enabling materials in mixed matrix membranes for high efficiency natural gas purification. Energy Environ. Sci..

[99] Bouma R.H.B., Checchetti A., Chidichimo G., Drioli E. (1997). Permeation through a heterogeneous membrane: The effect of the dispersed phase. J. Membr. Sci..

[100] Banhegyi G. (1986). Comparison of electrical mixture rules for composites. Colloid Polym. Sci..

[101] Dlubek G., Seidel A. (2008). Positron Annihilation Spectroscopy. Encyclopedia of Polymer Science and Technology.

[102] Dlubek G., Utracki L.A., Jamieson A.M. (2010). Local free-free volume distributions from PALS and dynamics of polymers. Polymer Physics: From Suspensions to Nanocomposites to Beyond.

[103] Liu M., Wong-Foy A.G., Vallery R.S., Frieze W.E., Schnobrich J.K., Gidley D.W., Matzger A.J. (2010). Evolution of nanoscale pore structure in coordination polymers during thermal and chemical exposure revealed by positron annihilation. Adv. Mater..

