# Supplementary material for: Correlation of Gas Permeability in a Metal-Organic Framework MIL-101(Cr)–Polysulfone Mixed-Matrix Membrane with Free Volume Measurements by Positron Annihilation Lifetime Spectroscopy (PALS)
Source: Membranes (Basel). 2013 Oct 25;3(4):331–53. doi: 10.3390/membranes3040331 (PMC4021949; doi:10.3390/membranes3040331)
Supplement: Supplementary File 1 — Supplementary Information (PDF, 1378 KB) [file membranes-03-00331-s001.pdf]

Supplementary Information

Instrumentation

X-ray diffraction analyses were carried out on a Bruker D2 Phaser using Cu $K\alpha_1/\alpha_2$ radiation with λ = 1.5418 Å.

Nitrogen sorption isotherms were measured at 77 K using a Quantachrome Autosorb iQ MP gas sorption analyzer. Ultra high purity (UHP, grade 5.0, 99.999%) nitrogen, and helium gases were used; the latter was used for performing cold and warm free space correction measurements. MIL-101 BET surface area (2694 m^2/g) and pore size were calculated using sample weights after degassing for 2 h at 120 °C with the built-in oil-free vacuum system of the instrument (ultimate vacuum < 10^{-8} mbar).

Thermogravimetric (TG) data were collected using a Netzsch Tarsus 209 F3 TGA instrument in a protecting flow of nitrogen (10 mL/min) at 10 °C/min heating rate.

Characterization (identification) of MIL-101

Figure S1. SEM image of MIL-101 microcrystals (Pd-coated, obtained on Zeiss Leo DSM 982 Gemini with field emitter).

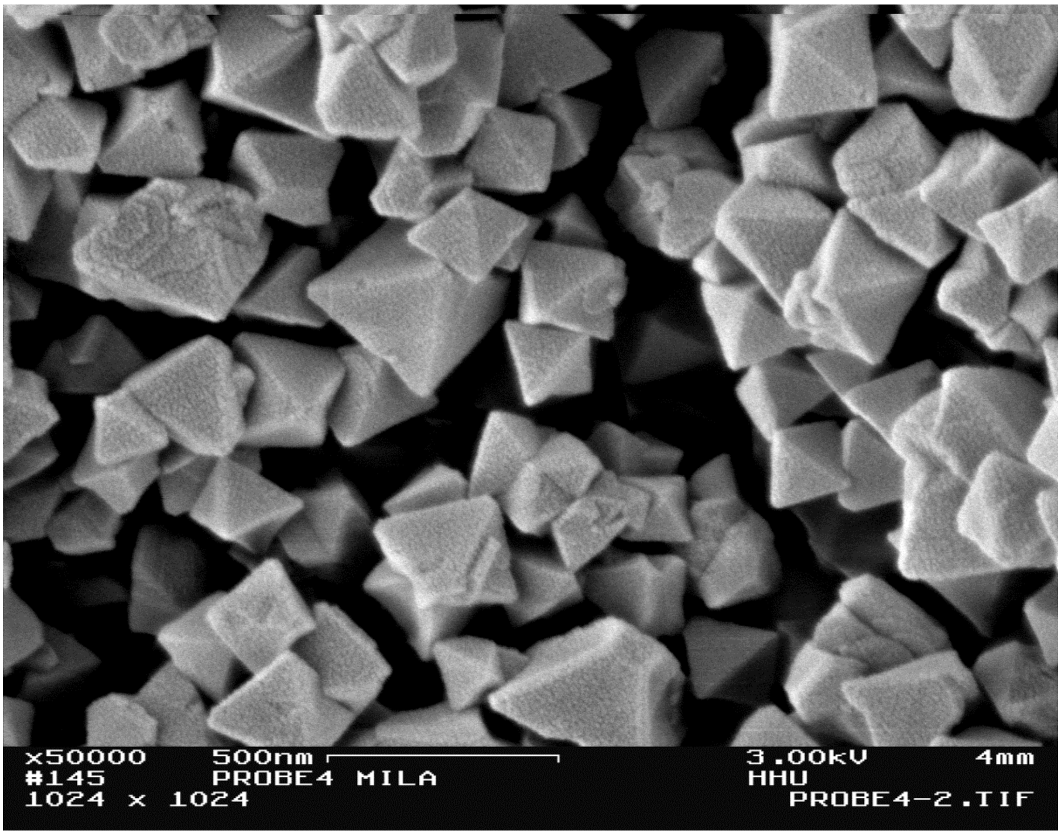

Figure S2. X-ray powder diffraction pattern of MIL-101(Cr), simulated from crystallographic cif-file; MIL-101(Cr), measured on activated (washed and dried) sample; MIL-101(Cr)/PSF mixed-matrix membrane with 24 wt % loading of MIL-101(Cr) and of polysulfone (PSF).

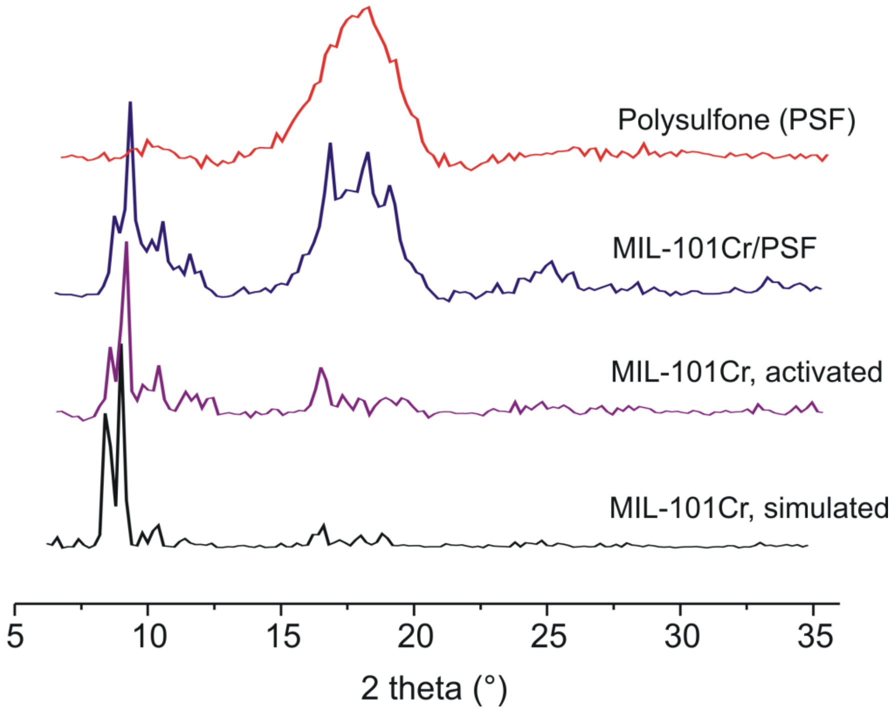

Figure S3. N_2 sorption isotherms of activated MIL-101(Cr). These sorption isotherms were fitted with the Brunauer–Emmett–Teller (BET) and Langmuir (L) equations to give S_{BET} (2690 m^2/g) and S_L (3630 m^2/g) surface areas and a total pore volume of 1.34 cm^3/g.

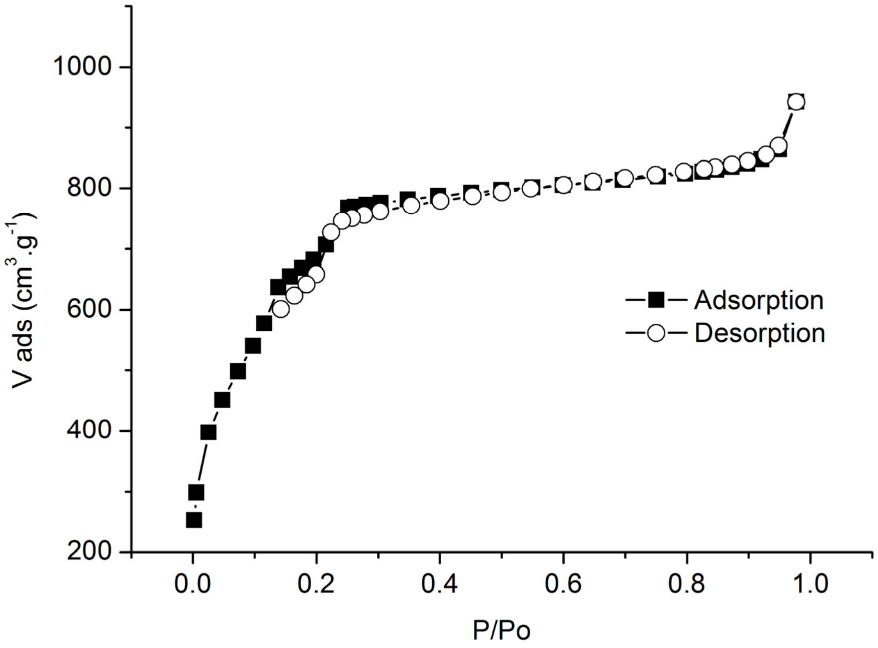

Figure S4. Thermogravimetric curves of as-synthesized MIL-101(Cr) (black), activated MIL-101(Cr) (red), 24 wt % activated MIL-101(Cr)/PSF mixed-matrix membrane (blue), and polysulfone (violet). (activated = the product was re-dispersed and centrifuged two times in DMF (20 mL) for 6 h, two timed in methanol (10mL) for 2 h and one time in water (10 mL) for 2 h. The final product was then dried at room temperature and ambient pressure).

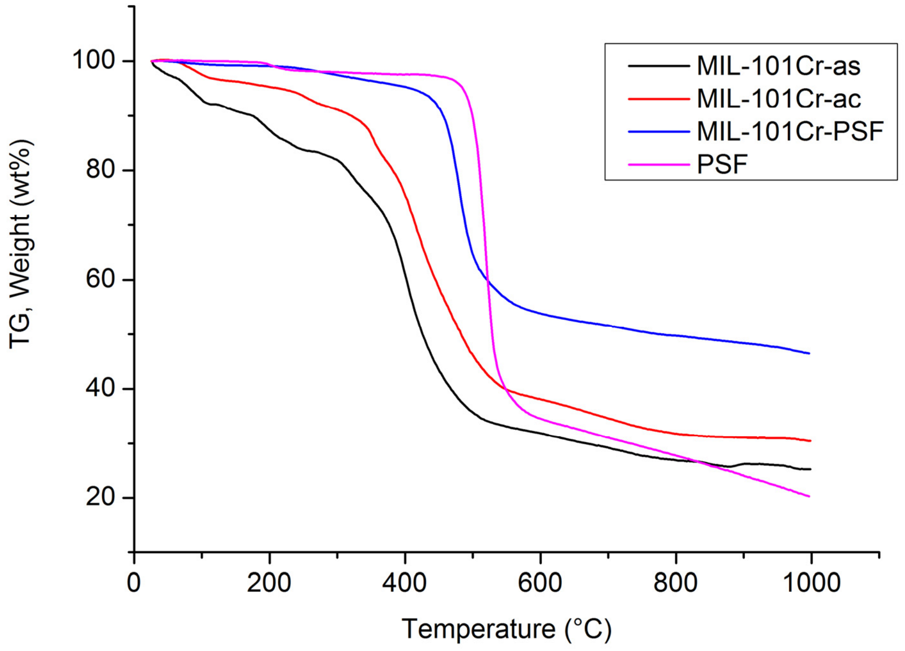

The weight loss for the activated sample between 120–350 °C indicates remaining terephthalic acid in the pores.

The unit barrer is a non-SI-unit in the cgs-system for the gas permeability of thin materials (in honor of the New Zealand chemist Richard M. Barrer (1910–1996), who was a leader in research on the diffusion of gases).

Permeability is defined to be the gas flow rate multiplied by the thickness of the material, divided by the area and by the pressure difference across the material. To measure this quantity, the barrer is the permeability represented by a flow rate of 10^{-10} cubic centimeters per second (volume at standard temperature and pressure, 0 °C and 1 atmosphere), times one centimeter of thickness, per square centimeter of area and centimeter of mercury difference in pressure. That is, 1 barrer = 10^{-10} $cm^2 \cdot s^{-1} \cdot cmHg^{-1}$ (Equation 1), or, in SI units, 7.5005 × 10^{-18} $m^2 \cdot s^{-1} \cdot Pa^{-1}$.

$$P(\text{barrer}) = 10^{-10} \frac{\text{cm}^3(\text{STP}) \cdot \text{cm}}{\text{cm}^2 \cdot \text{s} \cdot \text{cmHg}} \quad (1)$$

Modeling

Table S1 lists the relevant parameters used for the permeability and selectivity calculations according to the Maxwell model.

W_d and W_c are the weight and ρ_d and ρ_c the density of the dispersed filler and continuous polymer, respectively. The densities were taken from the literature.
W_c (PSF) = 400 mg;

density (ρ_c) (PSF) = 1.24 g/mL [1];
density (ρ_d) (MIL-101) = 0.62 g/mL [2].

P_c is the permeability of the continuous (pure) polymer phase.
P_c(PSF) for O_2 = 1.47 barrer;
P_c(PSF) for N_2 = 0.25 barrer;
Φ_d is the volume fraction of the dispersed phase.

Table S1. Correlation of MIL-101 wt % loading and filler volume fraction.

**MIL-101 wt %** [a]	**MIL-101 weight W_d (mg)**	**MIL-101 filler volume fraction (ϕ_d)**
0	0.000	0
7.5	32.432	0.1395
14	65.116	0.2456
19	93.827	0.3193
24	126.316	0.3871

[a] relative to polymer weight (W_c = 400 mg).

Figure S5. Correlation of MIL-101 wt % loading and filler volume fraction.

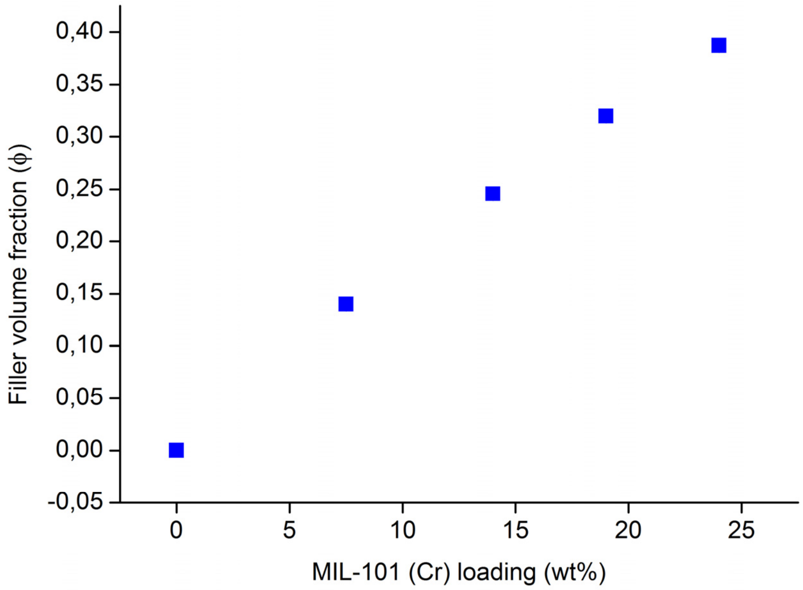

Table S2. CO_2/N_2 permeation results on MIL-101/PSF membranes, experiments performed at 30 °C and 3 bar total feed pressure (with standard deviations).

Membrane			P (CO_2) [a] [barrer]	P (N_2) [a] [barrer]	S (CO_2/N_2) [b]
Polymer amount	MIL-101(Cr) load (wt %)	Membrane Thickness (d) [μm]			
**300 mg**	***Pure polymer***	30.2	5.6 ± 0.6	0.29 ± 0.03	19.7 ± 3.9
**400 mg**	*7.5*	54.7	15.2 ± 1.6	0.63 ± 0.06	24.0 ± 4.8
	14	59.2	22.3 ± 2.2	0.84 ± 0.09	26.4 ± 5.3
	19	60.0	32.0 ± 3.3	1.20 ± 0.11	26.7 ± 5.4
	24	71.8	36.2 ± 3.6	1.30 ± 0.10	29.9 ± 6.0
**300 mg**	*7.5*	31.4	13.5 ± 1.4	0.56 ± 0.07	24.0 ± 4.7
	14	35.1	23.9 ± 2.5	1.21 ± 0.13	19.7 ± 4.0
	19	47.3	31.2 ± 3.2	1.21 ± 0.12	25.7± 5.2

[a] gas permeability; [b] ideal selectivity.

Table S3. CO_2/CH_4 permeation results on MIL-101/PSF membranes, experiments performed at 30 °C and 3 bar total feed pressure (with standard deviations).

Membrane			P (CO_2) [a] [barrer]	P (CH_4) [a] [barrer]	S (CO_2/CH_4) [b]
Polymer amount	MIL-101(Cr) load (wt %)	Membrane Thickness (d) [μm]			
**300 mg**	***Pure polymer***	30.2	5.6 ± 0.6	0.33 ± 0.04	16.9 ± 3.4
**400 mg**	*7.5*	54.7	15.2 ± 1.6	0.64 ± 0.05	23.9 ± 4.8
	14	59.2	22.3 ± 2.2	0.93 ± 0.10	24.0 ± 5.0
	19	60.0	32.0 ± 3.3	1.26 ± 0.13	25.3 ± 5.2
	24	71.8	36.2 ± 3.6	1.64 ± 0.20	22.2 ± 4.4
**300 mg**	*7.5*	31.4	13.5 ± 1.4	0.60 ± 0.05	22.7 ± 4.5
	14	35.1	23.9 ± 2.5	1.31 ± 0.12	18.3 ± 3.8
	19	47.3	31.2 ± 3.2	1.43 ± 0.15	21.9 ± 4.4

[a] gas permeability; [b] ideal selectivity.

Table S4. Gas (CO_2/N_2) permeation data of mixed-matrix membranes with MOFs from literature.

Polymer [a]	MOF	MOF (wt %)	P (CO_2) [b] [barrer]	P (N_2) [b] [barrer]	S [c] (CO_2/N_2)	Reference
Matrimid 5218	MOF-5	*0*	9.0	0.25	36.0	[3]
		10	11.1	0.28	39.6	
		20	13.8	0.40	34.5	
		30	20.2	0.52	38.8	
PSF	CuBTC	*0*	6.5	0.4	20	[4]
		5	7.7	0.3	25.1	
		10	7.9	1	8	
PSF	$Mn(HCOO)_2$	*0*	6.5	0.3	19.9	[4]
		5	6.5	0.3	19	
		10	6.8	0.2	26	
Matrimid 5218	CuBPY-HFS	*0*	7.29	0.22	33.1	[5]
		10	7.81	0.24	32.5	
		20	9.88	0.31	31.9	
		30	10.36	0.31	33.4	
		40	15.06	0.49	30.7	
Matrimid 5218	ZIF-8	*0*	9.52	0.31	30.7	[6]
		20	9.03	0.30	30.1	
		30	14.23	0.59	24.1	
		40	24.55	1.05	23.4	
		50	4.72	0.18	26.2	
		60	8.08	0.44	18.4	

[a] PVAC: poly(vinylacetate); PSF: polysulfone; [b] gas permeability; [c] all are ideal selectivities.

Figure S6. CO_2/N_2 Separation performance of MIL-101(Cr)/PSF mixed-matrix membranes, compared with the compiled data on MOF containing mixed-matrix membranes. Blue, pink and green points are the results for MIL-101(Cr)/PSF. The upper bound for polymer performances as defined by Robeson in 2008 [7] is shown.

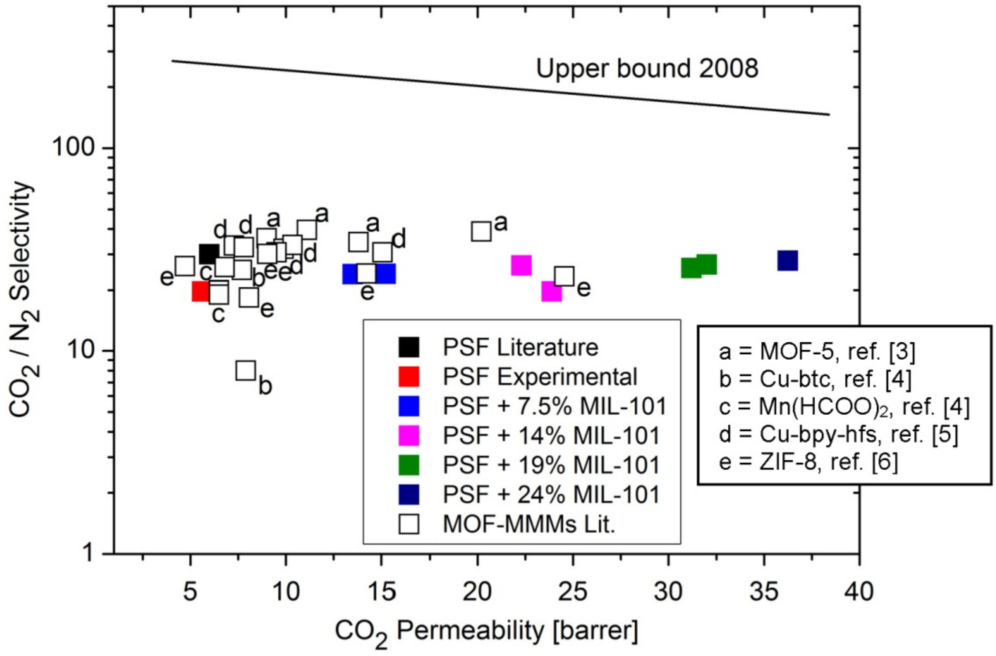

Table S5. Gas (CO_2/CH_4) permeation data of mixed-matrix membranes with MOFs from literature.

Polymer [a]	MOF	MOF (wt %)	P (CO_2) [b] [barrer]	P (CH_4) [b] [barrer]	S [c] (CO_2/CH_4)	Reference
Matrimid 5218	MOF-5	*0*	9.0	0.22	41.7	[3]
		10	11.1	0.22	51.0	
		20	13.8	0.34	40.5	
		30	20.2	0.45	44.7	
PSF	CuBTC	*0*	6.5	0.3	17	[4]
		5	7.7	0.3	22	
		10	7.9	1.3	7	
PSF	Mn(HCOO)$_2$	*0*	6.5	0.4	18	[4]
		5	6.5	0.5	16.5	
		10	6.8	0.8	9.5	
Matrimid 5218	CuBPY-HFS	*0*	7.29	0.21	34.7	[5]
		10	7.81	0.24	31.9	
		20	9.88	0.36	27.6	
		30	10.36	0.38	25.4	
		40	15.06	0.59	25.6	
Matrimid 5218	ZIF-8	*0*	9.52	0.24	39.8	[6]
		20	9.03	0.18	51.1	
		30	14.23	0.38	38.2	
		40	24.55	0.89	27.8	
		50	4.72	0.05	124.9	
		60	8.08	0.10	80.8	
Matrimid	ZIF-90	*0*	7.8	—	35.5	[9]
		15	12.5	—	35.6	
6FDA-DAM	ZIF-90B	*0*	400	—	17	
		15	680	—	26	
6FDA-DAM	ZIF-90A	*0*	400	—	17	
		15	800	—	27	
PSF	NH$_2$-MIL-53(Al)	*0*	4.7	0.2	23.5*	[10]
		8	4.7	0.13	29.3*	
		16	5.0	0.13	33.0*	
		25	5.4	0.10	46.0*	
		40	10.3	0.64	16.7*	
6FDA-ODA	UiO-66	*0*	14.4	0.33	44.1	[11]
		25	50.4	1.10	46.1	
6FDA-ODA	NH$_2$-UiO-66	*25*	13.7	0.27	51.6	[10]
6FDA-ODA	MOF-199	*25*	21.8	0.43	51.2	[10]
6FDA-ODA	NH$_2$-MOF-199	*25*	26.6	0.45	59.6	[10]
6FDA-ODA	UiO-67	*25*	20.8	1.40	15	[10]

[a] PVAC: poly(vinylacetate); PSF: polysulfone; 6FDA–DAM: 6FDA: 2,2-bis(3,4-carboxyphenyl) hexafluoro-propane dianhydride; DAM: diaminomesitylene; [b] gas permeability; [c] all are ideal selectivities except those marked with (*) which correspond to 50/50 % CO_2/CH_4 mixed gas selectivities; MOF-199 = CuBTC.

Figure S7. CO_2/CH_4 separation performance of MIL-101(Cr)/PSF mixed-matrix membranes, compared with the compiled data on MOF containing mixed-matrix membranes. Blue, pink and green points are the results for MIL-101(Cr)/PSF. The upper bounds for polymer performances as defined by Robeson in 1991 [12] and 2008 [7] are shown.

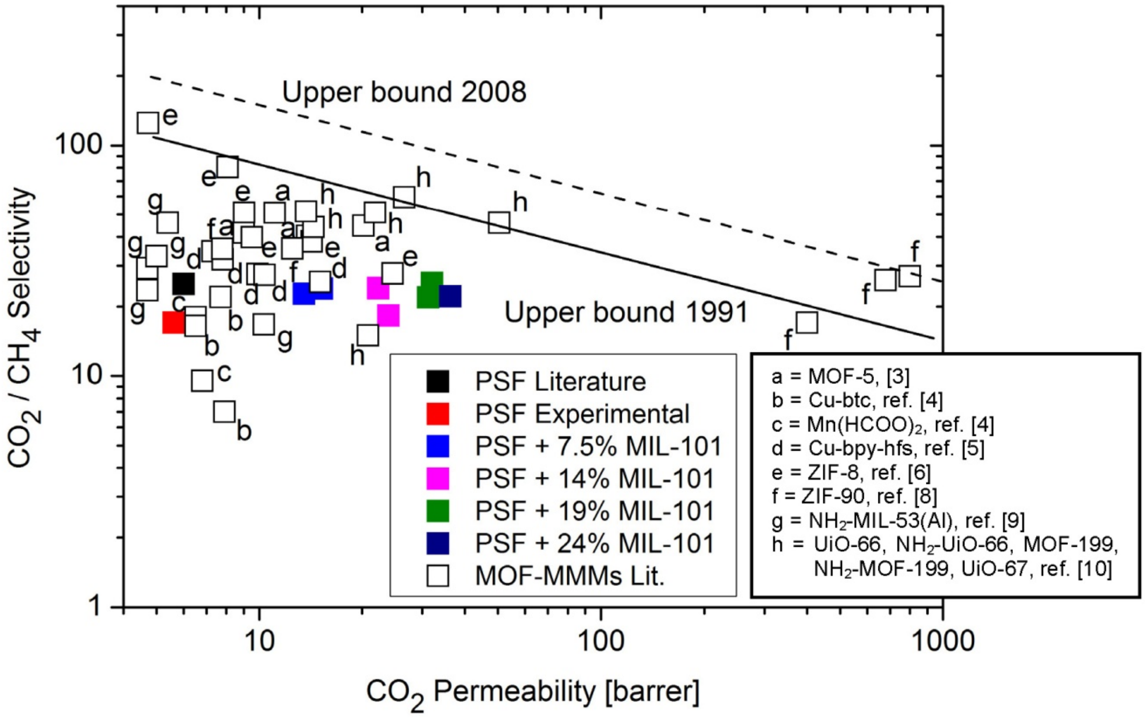

Table S6. Fitted o-Ps lifetimes and intensities for 5 and 6 components of pure MIL-101 respectively.

number of components	t_1 (ns)	t_2 (ns)	t_3 (ns)	t_4 (ns)	t_5 (ns)	t_6 (ns)	I_1 (%)	I_2 (%)	I_3 (%)	I_4 (%)	I_5 (%)	I_6 (%)
5 comp.	0.255	0.64	5.29	26.11	79.75	–	78.12	7.62	1.82	4.75	7.68	–
6 comp.	0.204	0.409	2.10	7.41	29.50	83.43	37.11	47.09	1.04	1.81	5.29	7.65

Table S7. Fitted o-Ps lifetimes and intensities for pure PSF and different MIL-101 loadings.

PSF-MIL-101 membranes	t_1 (ns)	t_2 (ns)	t_3 (ns) [a]	t_4 (ns)	I_1 (%)	I_2 (%)	I_3 (%)	I_4 (%)
Pure PSF	0.125	0.404	2.11	–	11.97	67.38	20.71	–
PSF + 7.5% MIL-101	0.125	0.393	2.06	8.3	10.77	68.78	18.78	1.66
PSF + 14% MIL-101	0.125	0.392	2.01	7.23	10.78	70.33	15.98	2.9
PSF + 19% MIL-101	0.125	0.393	2.02	7.29	10.69	71.15	15.07	3.09

[a] Lifetime t_3 for 6 component pure MIL-101 with 2.10 ns is too similar to t_3 = 2.11 ns for pure PSF and cannot be differentiated.

References

1. Product literature—Ultrason® (PESU, PSU, PPSU). Available online: http://www.plasticsportal.net/wa/plasticsEU~de_DE/portal/show/content/literature/ultrason (accessed on 18 October 2013).

2. Férey, G.; Mellot-Draznieks, C.; Serre, C.; Millange, F.; Dutour, J.; Surble, S.; Margiolaki, I. A chromium terephthalate-based solid with unusually large pore volumes and surface area. *Science* **2005**, *309*, 2040–2042.
3. Perez, E.V.; Balkus, K.J., Jr.; Ferraris, J.P.; Musselman, I.H. Mixed-matrix membranes containing MOF-5 for gas separations. *J. Membr. Sci.* **2009**, *328*, 165–173.
4. Car, A.; Stropnik, C.; Peinemann, K.V. Hybrid membrane materials with different metal-organic frameworks (MOFs) for gas separation. *Desalination* **2006**, *200*, 424–426.
5. Zhang, Y.; Musselman, I.H.; Ferraris, J.P.; Balkus, K.J., Jr. Gas permeability properties of Matrimid (R) membranes containing the metal-organic framework Cu-BPY-HFS. *J. Membr. Sci.* **2008**, *313*, 170–181.
6. Ordonez, M.J.C.; Balkus, K.J., Jr.; Ferraris, J.P.; Musselman, I.H. Molecular sieving realized with ZIF-8/Matrimid (R) mixed-matrix membranes. *J. Membr. Sci.* **2010**, *361*, 28–37.
7. Robeson, L.M. The upper bound revisited. *J. Membr. Sci.* **2008**, *320*, 390–400.
8. Bae, T.H.; Lee, J.S.; Qiu, W.L.; Koros, W.J.; Jones, C.W.; Nair, S. A high-performance gas-separation membrane containing submicrometer-sized metal-organic framework crystals. *Angew. Chem. Int. Ed.* **2010**, *49*, 9863–9866.
9. Zornoza, B.; Martinez-Joaristi, A.; Serra-Crespo, P.; Tellez, C.; Coronas, J.; Gascon, J.; Kapteijn, F. Functionalized flexible MOFs as fillers in mixed matrix membranes for highly selective separation of CO_2 from CH_4 at elevated pressures. *Chem. Commun.* **2011**, *47*, 9522–9524.
10. Nik, O.G.; Chen, X.Y.; Kaliaguine, S. Functionalized metal organic framework-polyimide mixed matrix membranes for CO_2/CH_4 separation. *J. Membr. Sci*. **2012**, *413–414*, 48–61.
11. Robeson, L.M. Correlation of separation factor versus permeability for polymeric membranes. *J. Membr. Sci.* **1991**, *62*, 165–185.

© 2013 by the authors; licensee MDPI, Basel, Switzerland. This article is an open access article distributed under the terms and conditions of the Creative Commons Attribution license (http://creativecommons.org/licenses/by/3.0/).
